# Supplementary material for: Quantum anomalous Hall crystals in moiré bands with higher Chern number
Source: Nat Commun. 2025 Jul 25;16:6875. doi: 10.1038/s41467-025-62224-9 (PMC12297688; doi:10.1038/s41467-025-62224-9)
Supplement: Supplementary file 1 — Supplementary Information [file 41467_2025_62224_MOESM1_ESM.pdf]

Supplementary Information for
‘Quantum anomalous Hall crystals in moiré bands with higher Chern number’

Raul Perea-Causin,[1] Hui Liu,[1] and Emil J. Bergholtz[1]

[1]*Department of Physics, Stockholm University, AlbaNova University Center, 106 91 Stockholm, Sweden*
Corresponding authors: raul.perea.causin@fysik.su.se, hui.liu@fysik.su.se, emil.bergholtz@fysik.su.se

Supplementary Note 1. Quasiparticle excitations in hole entanglement spectra

The hole entanglement spectrum typically consists of a low-eigenvalue sector located below an entanglement gap, where the number of low-eigenvalue states corresponds to the number of zero-energy quasiparticle excitations allowed by the system. Here, we detail the quasiparticle counting rules for the $\nu = \frac{2}{3}$ FCI in the $C = 1$ band and for the quantum anomalous Hall crystal in the $C = 2$ band. For the FCI case, the generalized exclusion rule restricts the system to no more than one hole in three consecutive orbitals. Such orbitals are given in our case by the 2D momenta folded into 1D, in analogy with the thin torus limit. This exclusion rule imposes the following constraint on the total quasiparticle excitations of the ground states [1],

$$N_{\text{qp}} = \frac{N_{\text{s}}(N_{\text{s}} - 2N_A - 1)!}{(N_{\text{s}} - 3N_A)!N_A!}, \tag{1}$$

where N_{s} is the number of orbitals (corresponding to the number of moiré sites in the finite system) and N_A is the number of holes in the A subsystem. When $N_A = 4$ and $N_{\text{s}} = 27$, this results in $N_{\text{qp}} = 5508$ below the first entanglement gap [Supplementary Figure 1(a)]. In contrast, for the case of quantum anomalous Hall crystals, the crystalline (CDW-like) order allows excitations corresponding only to the orbitals that are already occupied by a hole in each many-body ground state. The total number of configurations is therefore given by

$$N_{\text{qp}} = \frac{N_{\text{gs}}(N_{\text{s}} - N_{\text{e}})!}{(N_{\text{s}} - N_{\text{e}} - N_A)!N_A!}, \tag{2}$$

with N_{e} being the number of electrons and N_{gs} the number of degenerate ground states. For $N_A = 4$ and $N_{\text{s}} = 27$, this leads to $N_{\text{qp}} = 378$ [Supplementary Figure 1(b)], which is significantly fewer than in the FCI case. This sharp contrast in the number of states serves as a distinctive fingerprint to differentiate between crystalline orders and FCI phases. In addition, we note that the QAHC phase remains stable for smaller system sizes, cf. Supplementary Figure 1(c)-(d) and Supplementary Figure 2. Moreover, the K-point peaks in the structure factor become more pronounced with increasing system size. Concretely, we obtain $S(\mathbf{q} = \mathbf{K}) = 0.14, 0.16, 0.18$ for $N_{\text{s}} = 21, 24, 27$, i.e. the structure factor K-point peaks exhibit a linear dependence on the system size N_{s}, indicating the stability of the crystal structure in the thermodynamic limit.

Supplementary Note 2. Ground state momentum sectors

Here, we briefly explain how FCI and crystalline phases can be distinguished through the fact that the many-body ground states in these phases can be located at different momentum sectors. The center-of-mass momentum of a Fock state can be explicitly written as $\mathbf{k}_{\text{COM}} = \sum_{i \in \text{occupied}} \mathbf{k}_i$, where $\mathbf{k}_i$ denotes the i-th momentum and the sum is carried out over all occupied momentum states. For the $\nu = \frac{2}{3}$ FCI ground states, in the framework of thin-torus limit, the corresponding Fock states are $|011011011\cdots011\rangle$ (written in the occupation number representation in the momentum space folded into 1D, $|n_{\mathbf{k}_1}, n_{\mathbf{k}_2}, \cdots, n_{\mathbf{k}_{N_s}}\rangle$) and its two translation-invariant partners. In a finite-sized system with a geometry determined by the spanning vectors $\mathbf{R}_1$ and $\mathbf{R}_2$ (which define all possible momenta in the BZ), one can easily derive the concrete $\mathbf{k}_{\text{COM}}$ for all ground states. In a system with $\mathbf{R}_1 = (6, 3)$ and $\mathbf{R}_2 = (3, 6)$, the three FCI ground states are all located at the $\mathbf{\Gamma}$ momentum sector, while for $\mathbf{R}_1 = (6, 3)$ and $\mathbf{R}_2 = (1, 5)$ the three FCI ground states stay at the $\mathbf{\Gamma}$, $\mathbf{K}$, and $\mathbf{K}'$ momentum sectors as expected also from $\mathbf{K}$-point CDW order.

Supplementary Note 3. Fermi liquid in the ideal $C = 3$ band

As mentioned in the main text, the system exhibits a Fermi liquid behavior when for $\nu = \frac{2}{3}$ filling of ideal $C > 2$ bands. Here we provide numerical results for the $C = 3$ band and note that we observe similar physics in bands with higher Chern number. The system seems to have 6 ground states separated by a relatively large gap in the low-lying energy spectrum [see

Supplementary Figure 7(a)]. However, by threading a flux through the system the energy gap closes and all ground states now flow into excited states, as shown in Supplementary Figure 7(b). The absence of a gap together with the distribution of the occupation number of ground states shown in Supplementary Figure 7(c), which displays a clear Fermi surface (the occupation number of ground states changing from 1 to 0), indicates that this phase is a compressible Fermi liquid. Aligned with this, the ground-state pair-correlation function $G(\mathbf{r})$ is mostly constant away from the origin (i.e. away from the vertices in Supplementary Figure 7(d)), consistent with the liquid nature of this phase and further revealing the little impact of the moiré potential in this case.

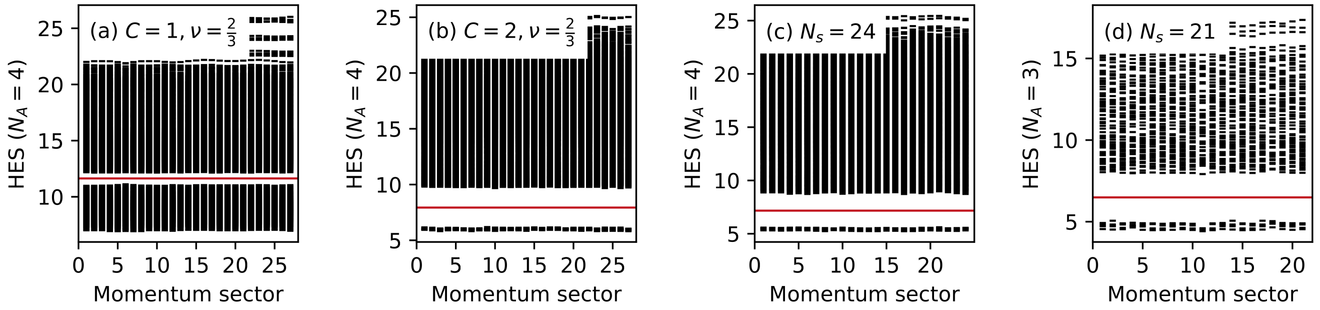

Supplementary Figure 1. Hole entanglement spectrum at filling $\nu = \frac{2}{3}$ of the (a) $C = 1$ and (b) $C = 2$ ideal Chern bands for system size $N_s = 27$ and $N_A = 4$. The number of states below the red line is 5508 in (a) and 378 in (b), matching the number of allowed quasiparticle excitations in the FCI and CDW (QAHC) orders, respectively. In (c) and (d) we show the HES in the $C = 2$ ideal Chern band for smaller system sizes $N_\mathrm{s} = 24$ and $N_\mathrm{s} = 21$, respectively, demonstrating the robustness of the QAHC phase even in small systems.

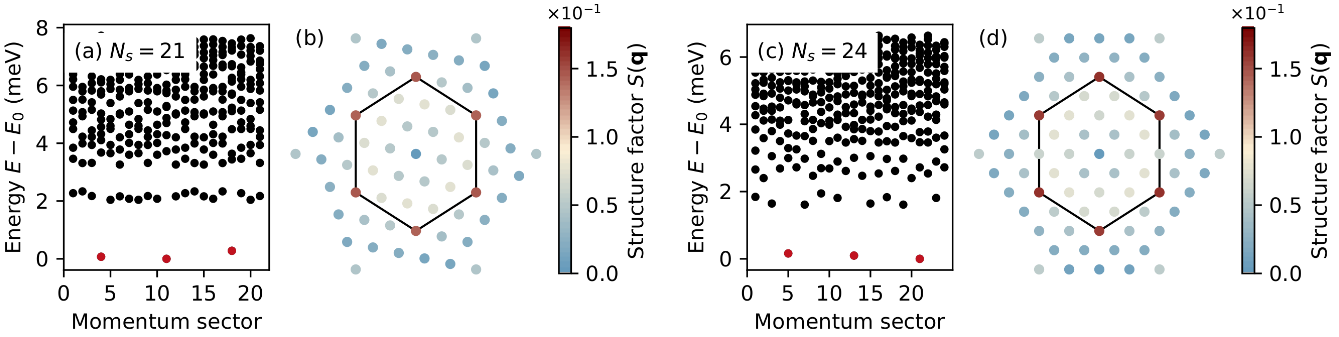

Supplementary Figure 2. Quantum anomalous Hall crystal at $\nu = \frac{2}{3}$ filling of the ideal $C = 2$ band for smaller system sizes. (a) Many-body spectrum displaying three nearly degenerate ground states and (b) structure factor characterized by $\mathbf{K}$-point peaks for a system with $N_\mathrm{s} = 21$ moiré sites. The analogous calculations for $N_\mathrm{s} = 24$ are shown in (c)-(d).

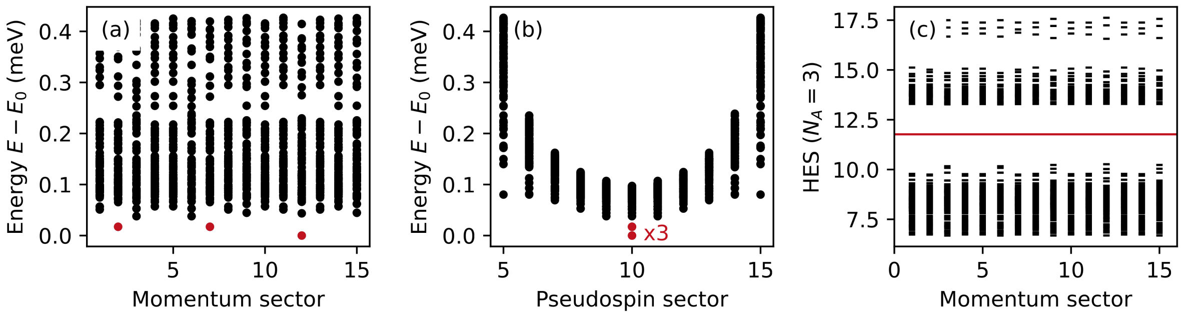

Supplementary Figure 3. Many-body energy spectrum for $\nu = \frac{2}{3}$ filling of two degenerate ideal $C = 1$ bands encoded in the (a) total momentum sectors and (b) total pseudospin sectors computed in a system with $N_s = 15$ sites and $N_e = 20$ electrons. Here pseudospin refers to the band degree of freedom. The average many-body Chern number of each ground state is $\mathcal{C}_{avg} = \frac{4}{3}$ indicating FCI order. (c) Hole-cut entanglement spectrum containing 3250 states below the first entanglement gap denoted by the red line. The same state counting was obtained in the particle-cut entanglement spectrum of the FCI emerging at $\nu = \frac{1}{3}$ filling of the $C = 2$ band in TDBG.

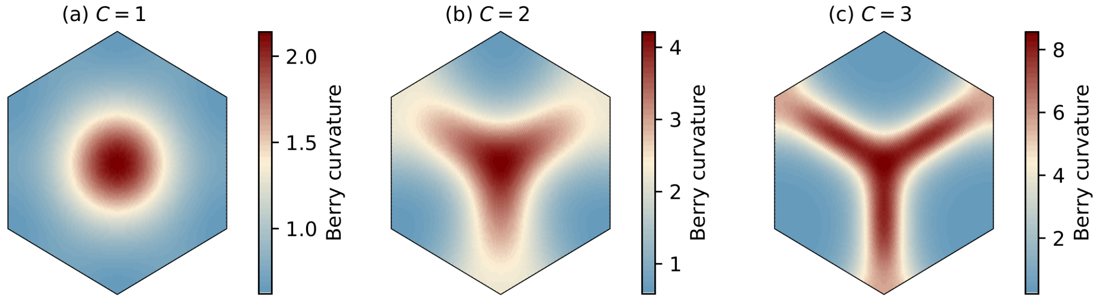

Supplementary Figure 4. Berry curvature of the single-particle flat bands in the chiral limit with Chern numbers (a) $C = 1$, (b) $C = 2$, (c) $C = 3$. The hexagons contain the moiré Brillouin zone (BZ). We plot the Berry curvature Ω normalized as $\Omega A_{BZ}/2\pi$, where A_{BZ} is the BZ area, so that the average across the BZ corresponds directly to the Chern number of the band.

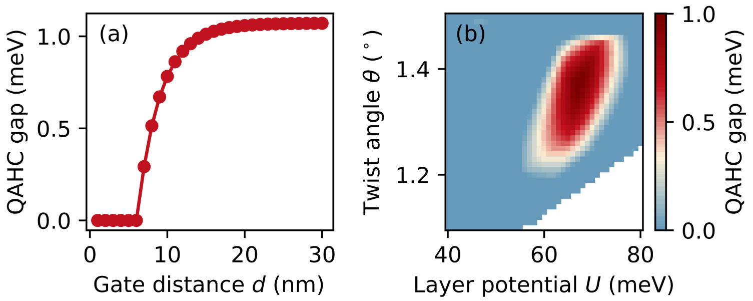

Supplementary Figure 5. Impact of gate-induced screening and layer-dependent interactions on QAHC phase in TDBG. (a) Energy gap between the highest QAHC many-body state and the lowest excited state as a function of the gate distance d considering the gate-induced interaction, $V(q) = \frac{e_0^2}{2A\epsilon\epsilon_0 q}\tanh(qd)$. Here the parameters $(U, \theta) = (60\text{ meV}, 1.35°)$ in the TDBG system are taken. (b) QAHC gap as a function of twist angle and layer potential considering layer-dependent interactions between electrons in layers l and l', $V_{ll'}(q) = \frac{e_0^2}{2A\epsilon\epsilon_0 q}\exp(-qd_g|l-l'|)$, where $d_g = 0.334$ nm is the separation between nearest graphene layers. In all calculations we considered $N_e = 14$ electrons in $N_s = 21$ sites.

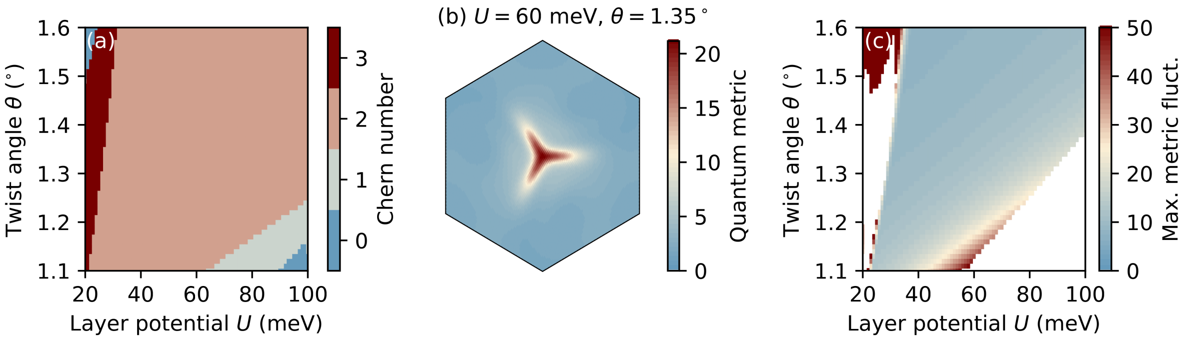

Supplementary Figure 6. Quantum geometric properties of the single-particle conduction band in twisted bilayer graphene. (a) Chern number of the band as a function of twist angle θ and layer potential U. (b) Quantum (Fubini-Study) metric distribution in the BZ for $U = 60$ meV, $\theta = 1.35°$. (c) Maximum fluctuation of the quantum metric in the BZ as a function of (U, θ). The white regions correspond to the band not being isolated, i.e. where the indirect band gap is zero. The quantum metric is normalized as $g_{\mathbf{k}} A_{\mathrm{BZ}}/2\pi$.

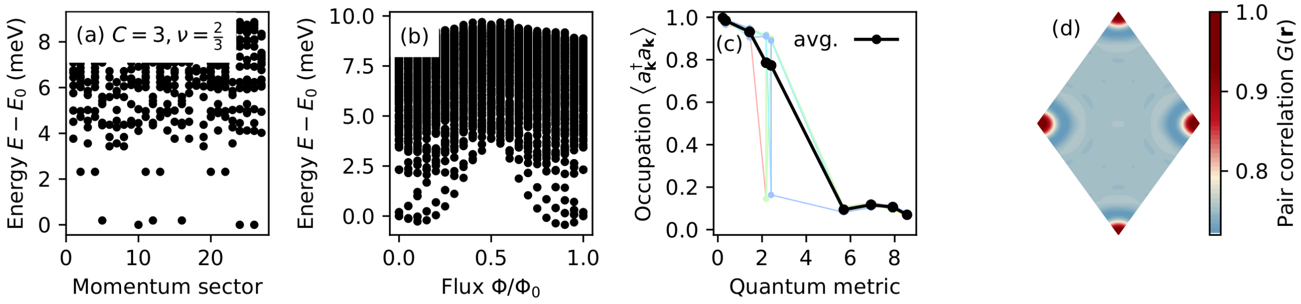

Supplementary Figure 7. Compressible liquid in the ideal $C = 3$ system at $\nu = \frac{2}{3}$ filling. (a) Many-body energy spectrum with six nearly degenerate ground states. (b) Spectral flow showing the absence of a gap in the many-body spectrum. (c) Many-body occupation $\langle a^{\dagger}_{\mathbf{k}} a_{\mathbf{k}} \rangle$ as a function of the single-particle quantum metric $g(\mathbf{k}) A_{\mathrm{BZ}}/2\pi$ which is normalized with respect to the BZ area. The thick black line is the average over the six ground states, while each individual contribution is color-coded. The occupation shows a behaviour reminiscent of the Fermi distribution. The metric $g(\mathbf{k})$ throughout the Brillouin zone is shown in Supplementary Figure 4(c). (d) Pair-correlation function $G(\mathbf{r})$ showing the liquid character of this phase.

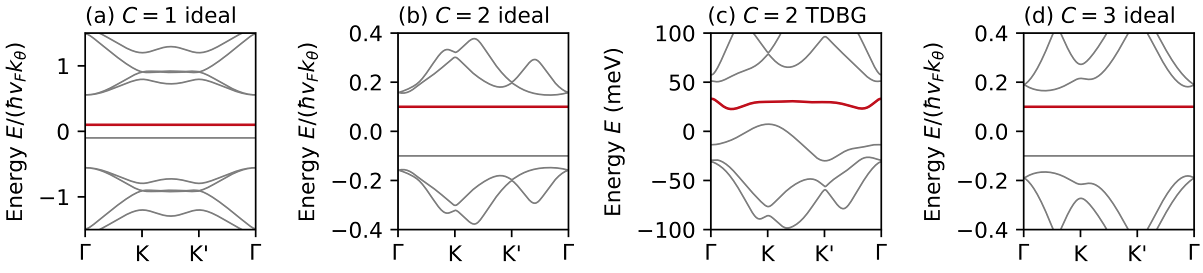

Supplementary Figure 8. Band structures of the considered systems. (a) Chiral model of twisted bilayer graphene displaying an ideal $C = 1$ flat band, (b) chiral model of twisted bilayer–trilayer graphene displaying an ideal $C = 2$ flat band, (c) realistic model of twisted double bilayer graphene displaying a nearly flat $C = 2$ band, and (d) chiral model of twisted trilayer–trilayer graphene displaying an ideal $C = 3$ flat band. The target bands are marked in red.

Supplementary references

[1] N. Regnault and B. A. Bernevig, Fractional Chern insulator, Phys. Rev. X **1**, 021014 (2011).
